# Social prescribing interventions in response to older adults’ needs in community settings – nurses’ perspectives: a qualitative study

**DOI:** 10.1177/17449871261458631

**Published:** 2026-08-05

**Authors:** Rute Sadio, Adriana Henriques, Paulo Nogueira, Andreia Costa

**Affiliations:** Primary Health Care Nurse and Researcher, Nursing Research Innovation and Development Centre of Lisbon (CIDNUR), School of Nursing, Universidade de Lisboa, Lisbon, Portugal; ULSAC – Unidade Local de Saúde do Alentejo Central, UCSP Estremoz, Estremoz, Portugal; Professor and Researcher, Nursing Research Innovation and Development Centre of Lisbon (CIDNUR), School of Nursing, Universidade de Lisboa, Lisbon, Portugal; Instituto de Saúde Ambiental (ISAMB), Faculty of Medicine, University of Lisbon, Lisbon, Portugal; Laboratório para a Sustentabilidade do Uso da Terra e dos Serviços dos Ecossistemas – TERRA, Lisbon, Portugal; Professor and Researcher, Nursing Research Innovation and Development Centre of Lisbon (CIDNUR), School of Nursing, Universidade de Lisboa, Lisbon, Portugal; Instituto de Saúde Ambiental (ISAMB), Faculty of Medicine, University of Lisbon, Lisbon, Portugal; Laboratório para a Sustentabilidade do Uso da Terra e dos Serviços dos Ecossistemas – TERRA, Lisbon, Portugal; Professor and Researcher, Nursing Research Innovation and Development Centre of Lisbon (CIDNUR), School of Nursing, Universidade de Lisboa, Lisbon, Portugal; Instituto de Saúde Ambiental (ISAMB), Faculty of Medicine, University of Lisbon, Lisbon, Portugal; Laboratório para a Sustentabilidade do Uso da Terra e dos Serviços dos Ecossistemas – TERRA, Lisbon, Portugal

**Keywords:** aged, community health nursing, health promotion, primary healthcare, social determinants of health, social prescribing

## Abstract

**Background:**

Social prescribing is a community-based strategy to address older adults’ needs. In primary healthcare, nurses are key in linking individuals with community resources, but evidence on their implementation of this practice is limited.

**Aims:**

To explore primary healthcare nurses’ perspectives on effective social prescribing interventions using the Fundamental Care Model.

**Methods:**

A descriptive qualitative study was conducted with nurses through focus groups. Data were analysed using thematic analysis and interpreted through the relational, integrative and contextual dimensions of the Fundamental Care Model.

**Results:**

Social prescribing was seen as a relational practice based on trust. Group physical activity, health literacy and cultural activities effectively promoted autonomy and social participation. Key barriers included transportation issues, limited consultation time and uneven resource availability.

**Conclusion:**

Social prescribing is a viable strategy to support older adults’ autonomy. This study operationalises the Fundamental Care Model within community nursing practice and provides evidence to inform nursing-led social prescribing strategies and health policy integration through organisational support and intersectoral collaboration.

## Introduction

Primary healthcare (PHC) refers to the first level of contact within health systems, where care is delivered close to people’s communities and everyday environments. It is characterised by accessibility, continuity and coordination of care and plays a central role in promoting health, preventing disease and managing chronic conditions (Roberti et al., 2025). In PHC settings, care provision extends beyond clinical treatment to include links with community resources and attention to social determinants of health (Frieden et al., 2023). Effective coordination of care, involving collaboration between professionals, services and sectors, is essential to ensure continuity and quality in responding to population needs (Khatri et al., 2023). In this context, nurses working in PHC are key professionals, acting as community-based practitioners with a comprehensive view of the individual (Husk et al., 2020) and the ability to establish therapeutic relationships that support an integrated approach to users’ needs (Ribeiro et al., 2024; Younas et al., 2023).

People accessing PHC, including older adults, often present complex and multifactorial needs (Costa et al., 2024), encompassing physical, emotional, social and relational dimensions (Frieden et al., 2023). In this study, the term older adults refers to individuals aged 65 years and over, consistent with international public health definitions of ageing populations (United Nations, 2022). This population group was selected due to the increased prevalence of chronic conditions, social isolation, functional decline and complex psychosocial needs, which frequently require integrated and community-based responses (Ballmer and Gantschnig, 2024; Al-Habbal et al., 2025; Sherman et al., 2024). The presence of chronic conditions, persistent pain, loss of mobility and functionality decline requires interventions aimed at promoting autonomy and alleviating symptoms (Ballmer and Gantschnig, 2024; Hafiz et al., 2024). At the same time, factors such as social isolation, loneliness, unemployment, grief and stress have a direct impact on health and well-being (Sadio et al., 2025) and often remain unaddressed without a holistic assessment (Sherman et al., 2024). These needs are intrinsically linked to the social determinants of health, including living conditions, access to support networks, educational level and community integration (Al-Habbal et al., 2025).

In this context, social prescribing has emerged as an innovative strategy that enables health professionals to refer individuals to a range of non-clinical, community-based interventions such as walking or gardening groups, swimming programmes, cultural initiatives, emotional support groups or volunteering (Kurpas et al., 2023; Menhas et al., 2023; Morse et al., 2022; Wilson et al., 2025). These interventions have the potential to simultaneously address physical needs (such as pain management or maintaining mobility), emotional needs (such as psychological well-being and self-esteem) and social needs (such as a sense of belonging and integration) (Islam, 2025; Scarpetti et al., 2024). In addition, social prescribing can function as mechanisms to support the balance between individual resources and environmental challenges, promoting the maintenance of autonomy and a sense of identity (Ballmer and Gantschnig, 2024). Emerging evidence suggests that social prescribing contributes to improvements in well-being, social connectedness and self-management, particularly among individuals with complex health and social needs (Husk et al., 2020; Islam, 2025; Morse et al., 2022). However, the existing evidence base remains heterogeneous, with variations in intervention design and outcome measures, which can limit the strength of conclusions regarding its effectiveness and long-term impact (Husk et al., 2020; Morse et al., 2022). The practice of social prescribing is especially relevant in vulnerable populations, particularly older adults, who often experience accumulated physical and psychosocial frailties (Mansell et al., 2024).

However, the effectiveness and sustainability of this approach depend on several factors (Frieden et al., 2023), including the quality of the relationship between professional and user, the capacity for personalised assessment, the availability of community resources and organisational and political-institutional support for interdisciplinary practice (Roberti et al., 2025). Previous research suggests that relational continuity, trust and tailored support play a key role in shaping engagement with social prescribing interventions, particularly in PHC contexts (Tierney et al., 2020). In this context, nurses, due to their proximity to patients, knowledge of the community and competence in comprehensive assessment of individuals, play a strategic role in identifying needs and implementing social prescribing interventions (Carrera-Noguero et al., 2025; Younas et al., 2023). Recent reviews and integrative reviews further emphasise that nursing involvement is crucial for the success of these programmes, particularly in bridging the gap between clinical care and community-based support (Husk et al., 2020; Morse et al., 2022; Tierney et al., 2020).

Despite the growing international recognition of social prescribing, there is limited empirical evidence exploring how nurses conceptualise and operationalise this practice within PHC settings, particularly from a theoretically grounded perspective. This understanding is fundamental for developing sustainable practices adapted to real-world contexts, improving community referral processes and strengthening the capacity of PHC to respond to needs that transcend the biomedical domain (Simonelli et al., 2025; Spanos et al., 2025). Furthermore, it contributes to the consolidation of social prescribing as an integrated, evidence-based approach to promoting health and quality of life (Fleming, 2023). Given the complexity of these needs, which simultaneously involve physical, emotional and social components, the use of theoretical frameworks becomes essential to support understanding and guide person-centred practices (Feo and Kitson, 2016). To address this complexity, the study is informed by the Fundamental Care Model (Kitson et al.,2013), which conceptualises care across three interdependent dimensions: (1) the therapeutic relationship between professional and user; (2) the integration of physical, emotional and social care; and (3) the organisational, cultural and political context in which care occurs. Internationally, this type of care continues to be recognised as undervalued and often invisible, making it essential to reinforce its value and ensure its systematic integration into health systems (Kitson et al., 2019). This conceptual framework aligns with the synthesis presented by Khatri et al. (2023), which describes the continuity and coordination of care in PHC across three analytical levels: individual, organisational, and systemic, highlighting the importance of coordination between these levels to ensure integrated and sustainable responses.

This model provides a robust conceptual framework for analysing how nurses incorporate social prescribing into their daily practice, how it responds to the multiple needs of users, and what factors influence its application in a community context (Carrera-Noguero et al., 2025; Muhl et al., 2023).

In this context, the present study aims to explore PHC nurses’ perspectives on social prescribing interventions considered effective in addressing older adults’ needs within community settings, using the Fundamental Care Model as an interpretative framework.

## Method

### Study design and theoretical framework

A qualitative descriptive approach was adopted, informed by a social constructionist perspective, in which knowledge is understood as a product of social interactions, cultural contexts and discursive practices (Burr, 2024; Charmaz, 2014). This approach was considered appropriate for exploring how nurses conceptualise and integrate social prescribing into everyday practice, as it prioritises participants’ experiences, meanings and interpretations within context. The qualitative descriptive design also allows the generation of rich data grounded in participants’ concrete experiences and supports the exploration of multiple perspectives within a shared social context, which is particularly relevant to PHC settings where clinical, social and community dimensions intersect (Creswell, 2013).

These characteristics are fundamental to capturing the complexity inherent in the work of nurses in PHC, where clinical, social and community dimensions are intertwined.

Data analysis was guided by the Fundamental Care Model (Kitson et al., 2013), which conceptualises care across three interdependent dimensions: the therapeutic relationship, integration of physical, emotional and social care, and the organisational and socio-political context. This framework provided a theoretically grounded lens for interpreting nurses’ perceptions of social prescribing.

The research team had previous experience in qualitative methodologies and in studies with adult and older adult populations. The principal investigator had professional experience in PHC and qualitative research and received supervision from senior researchers. This experience positively contributed to the quality of data collection and analysis.

### Participants

The study was conducted in PHC units within a regional health system. These units included Community Care Units, which provide community-based and home care services; Family Health Units, which deliver multidisciplinary primary care services; and Personalised Health Care Units, which focus on individualised primary care provision.

Intentional sampling was used, planned to include between 8 and 10 participants per focus group (Creswell, 2013). Participants were recruited through institutional contacts within the health units, and invitations were sent by email to nurses meeting the inclusion criteria, namely working in PHC and having experience in community-based care. Eighteen nurses were invited and all agreed to participate.

The participants worked across different health units and had diverse professional backgrounds, with most holding specialist qualifications in areas such as community and public health, rehabilitation, medical-surgical, maternal and obstetric, health, and mental and psychiatric health. They were involved in daily clinical activities with direct contact with users and families.

The groups were organised according to participants’ availability, ensuring diversity in terms of gender, age, length of professional experience and areas of expertise (Table 1). Considering the low representation of male nurses in PHC, all male professionals who expressed availability were included. All participants were informed about the study objectives and provided written informed consent prior to participation.

**Table 1.** Socio-demographic characteristics of focus group participants.

Nursing specialty	Value	Years of professional experience	Value
Community/Public Health Nursing	7	⩽10 years	2
Rehabilitation Nursing	2	11–20 years	2
Mental Health and Psychiatric Nursing	1	20–30 years	11
Maternal and Obstetric Health Nursing	1	>30 years	3
No specialty	7		

### Data collection

Data collection was carried out in September 2025 through two focus groups, each with nine participants. Focus groups were chosen as they enable the exploration of shared experiences, interaction between participants and the co-construction of meanings, which is particularly relevant when examining professional perspectives within a common practice context (Guest et al., 2020). This method allowed participants to reflect collectively on social prescribing practices, generating richer data than individual interviews. The number of focus groups was considered adequate to address the study aim, as data saturation was achieved, with no substantially new themes emerging in the second group.

The first focus group took place in person at a PHC facility and the second online, both in a private setting, ensuring privacy and promoting open sharing among participants. Each session lasted approximately 90 minutes and was moderated by the principal investigator, conducted in line with methodological recommendations for focus groups (Guest et al., 2020). Although the researcher had a prior professional relationship with participants as colleagues in PHC, reflexive strategies and external supervision were used to minimise potential bias. Only the researcher and participants were present at each session.

A semi-structured guide (Table 2) was developed based on the literature on social prescribing and was pre-tested with two PHC nurses who were not included in the study, to assess clarity, relevance and flow of the questions. Minor adjustments were made following this process. The guide addressed: (1) perceived needs in older adults; (2) previous experiences with social prescribing; (3) community activities considered most impactful; (4) the role of nurses in referral and follow-up, and (5) barriers and facilitators to implementation.

**Table 2.** Focus group interview guide.

Guiding topic	Focus group questions
1. Needs of older adults	In your experience, what are the main needs of older adults in the community where you work?
2. Experiences with social prescribing	Have you been involved in any intervention or activity that could be considered social prescribing? Can you share examples?
3. Interventions with impact	What types of community interventions (activities, groups or programmes) do you consider to make a real difference in older adults’ well-being and quality of life?
4. Nurses’ involvement	What has been your role, as nurses, in identifying or referring older adults to this type of intervention?
5. Facilitators and barriers	What makes it easier or more difficult to integrate social prescribing into the care you provide?

The discussions were audio-recorded and transcribed in full. After each session, the researcher prepared field notes to record relevant nonverbal elements, such as facial expressions, intonation or emotional reactions. The transcripts were then returned to the participants (member checking), who confirmed the accuracy of the records.

### Data analysis

The focus group recordings were transcribed in full and analysed using MAXQDA software, following a rigorous and systematic process in accordance with international standards for qualitative research (Creswell, 2013; Velloso and Tizzoni, 2020). Data were analysed using thematic analysis, guided by the Fundamentals of Care Framework (Kitson et al., 2013), which was used as a deductive lens to support the initial coding and organisation of the data.

The analysis followed three complementary stages: (1) detailed reading of the transcripts to familiarise with the data; (2) coding of relevant excerpts, combining deductive coding informed by the theoretical framework with inductive coding to capture emerging themes and (3) in-depth interpretation to identify patterns, relationships and underlying meanings in participants’ experiences.

Any discrepancies between researchers’ interpretations during the coding process were discussed until consensus was reached, and all material was subsequently reviewed and validated by the co-authors to ensure methodological consistency. The use of representative quotations contributed to the transparency and credibility of the analysis (Braun and Clarke, 2021). After the second focus group, data saturation was verified, understood as the point at which no new relevant information emerged, confirming the adequacy of the sample.

### Rigour, reflexivity and ethical considerations

The quality and reliability of the study were ensured by applying the criteria of Lincoln and Guba (1985). Credibility was reinforced by the diversity of participants and the triangulation of researchers, following contemporary recommendations for methodological robustness (Noble and Smith, 2015). Transferability was ensured by a detailed description of the context, sample and methodological procedures. Reliability and confirmability were ensured by maintaining an audit trail recording methodological and analytical decisions.

The study was reported in accordance with the Consolidated Criteria for Reporting Qualitative Research (COREQ) checklist (Tong et al., 2007; see Supplemental Material 1). Reflexivity was promoted through reflective records kept by the principal investigator, which allowed for the identification and mitigation of potential biases related to her professional experience as a nurse. This practice contributed to clarifying the researcher’s position and reinforcing the transparency of the analytical process.

This study was approved by a Regional Health Administration Ethics Committee (Approval No. XX/CE/2022) and conducted in accordance with the Declaration of Helsinki (1975). Data confidentiality was guaranteed, ensuring anonymity and protection of the participants’ identity at all stages of the study.

## Results

A total of 18 PHC nurses participated in the study, with diverse professional backgrounds and experience in community-based care. Most participants had specialist training in different areas of nursing and were involved in direct clinical practice with individuals and families. Although social prescribing is not a formally established role in this context, participants reported experience in identifying social needs and referring individuals to community-based resources as part of their routine practice.

The results describe how nurses recognise and implement social prescribing interventions in response to older adults’ needs in the community context. The dimensions and subthemes identified resulted from the analysis of responses to the five guiding questions of the focus group guide, constructed based on the Fundamentals of Care Framework (Table 3). This framework enabled the organisation of nurses’ perceptions into three interdependent dimensions: Relational, Integration, and Contextual; reflecting social prescribing interventions, professional roles and the factors influencing their implementation in community settings. Table 4 presents the themes and subthemes identified according to the Fundamentals of Care Framework.

**Table 3.** Correspondence between questions in the guide and subtopics identified according to the Fundamentals of Care Framework.

Guiding questions from the script	Empirical evidence in the results	Dimension/corresponding subtopics
1. Needs felt by the elderly population	Isolation, loneliness, lack of safe spaces, transportation difficulties	Relational: Monitoring and proximity Contextual: Transportation barriers
2. Experiences with social prescription	Participation in community groups, physical and cultural activities, involvement of nurses in initiatives	Integration: Group physical activity; Appreciation of local culture and traditions Relational: Trust and motivation
3. Interventions with impact	Health literacy groups, workshops and intergenerational programmes	Integration: Health literacy; cultural appreciation
4. Nurse involvement	Relationship of trust, motivation, personalised support and role as facilitator	Relational: Trust, motivational role and closeness
5. Facilitators and barriers	Lack of time and resources, transportation barriers, support from local authorities and senior universities	Contextual: Available resources and time; support from local institutions

**Table 4.** Themes and subthemes identified according to the Fundamentals of Care Framework.

Dimension	Theme	Subthemes
1. Relational	Therapeutic relationships	Trust in nurses; motivational role; continuity and proximity of care
2. Integration	Integrated physical, emotional and socio-cultural care	Group physical activity; health literacy; appreciation of local culture and traditions
3. Contextual	Organisational and community conditions	Transportation barriers; available resources and time; support from local institutions

### Relational

The analysis of this dimension revealed three interrelated subthemes: trust, the motivational role of nurses, and continuity and proximity of care, which structure the therapeutic relationship and support adherence to social prescribing interventions in everyday care.

#### Older adults’ trust in nurses

The nurse–older adult relationship emerged as a structuring axis of social prescribing, constituting the foundation on which adherence and participation in community activities are built. Trust was described by participants as the indispensable starting point for involvement in the proposed initiatives. One of the nurses summarised this perception by stating: ‘Nurses are seen as trustworthy figures, which gives them influence . . .’ (Participant 1). Participants also highlighted that the absence of regular follow-up care may weaken this relationship, as illustrated in the second focus group ‘. . .Many older adults in Family Health Units do not receive regular follow-up care beyond their appointments, and loneliness is a widespread reality’ (Participant 2). This finding suggests that, in some cases, the trust established during consultations becomes the primary link maintaining older adults’ connection with the healthcare system, particularly in contexts where ongoing support is limited.

#### The motivational role of nurses

Closely linked to the therapeutic relationship, nurses’ motivational role emerged as a key driver for the active involvement of older adults in community-based initiatives. Participants’ statements illustrate a proactive and encouraging approach: ‘Whenever I can, I emphasise to users the importance of participating in group physical activities’ (Participant 5). This statement illustrates that relational bonds translate into encouragement and persistence in reinforcing adherence to activities. Participant 5 added a broader perspective to the motivational role, stating: ‘Community health is not only about preventing disease, but also creating opportunities for socialisation’. In this sense, social prescribing reinforces nursing’s long-standing role in promoting community ties, prevention, and social integration, extending care beyond a predominantly biomedical focus. Motivation is described not only as verbal encouragement but also as ongoing and personalised follow-up, supported by the therapeutic relationship developed over time.

#### Continuity and proximity of care

Proximity and ongoing support emerge as natural extensions of the therapeutic relationship. Telephone contact and regular follow-up were identified as effective strategies for reducing social isolation, with continuity of the relationship being highlighted as a determining factor, even at a distance: ‘Telephone follow-ups maintain proximity and help encourage participation’ (Participant 7). This seemingly simple practice illustrates how essential care extends beyond face-to-face consultations, mitigating the risk of social isolation. In the same vein, Participant 8 emphasised the importance of creating safe spaces for social interaction, stating: ‘Many older adults miss safe spaces where they can socialize and feel useful’.

From this perspective, nurses assume the role of relational facilitators, creating environments that promote trust, belonging and motivation for participation. Such practices demonstrate that commitment to well-being extends beyond the formal setting of consultations, integrating emotional and social dimensions and reinforcing nurses as key agents of relational care.

### Integration

Three areas of integrated intervention emerged: group physical activity, health literacy, and appreciation of local culture and traditions, through which social prescribing is operationalised in coordinated responses to the physical, emotional, and socio-cultural needs of older adults.

#### Group physical activity

Physical and expressive activities were described as privileged ways of promoting physical well-being, social interaction and a sense of belonging. Nurses referred to the importance of physical exercise groups, such as community walks and water aerobics, also highlighting the integrative role of activities that combine movement and expression, such as senior dance or theatre: ‘Gymnastics, dance, or even theatre are very helpful, not only for physical reasons, but because they encourage seniors to socialize and feel less lonely’ (Participant 6).

One of the participants pointed out ‘. . .the promotion of physical exercise groups, such as outdoor walks or dancing’ (Participant 7), whereas Participant 8 added that ‘Even simple activities, such as group rehabilitation exercises or gymnastics, give older adults a sense of belonging and capability’. These interventions reveal that physical practices, in addition to their physical benefits, take on a symbolic and relational role, transforming physical movement into experiences of belonging, recognition and personal appreciation.

#### Health literacy

Health literacy has been widely recognised as the foundation for autonomy, enabling older adults to understand and manage their own health conditions. One participant highlighted the importance of ‘. . .health literacy groups, either general or dedicated to specific conditions’ (Participant 7). This dimension broadens the educational role of nurses and reinforces their function of promoting autonomy and individual empowerment. Similarly, Participant 9 emphasised that these initiatives are ‘. . .essential, especially for managing chronic diseases’, highlighting its contribution to self-care and informed health management.

#### Appreciation of local culture and traditions

The appreciation of culture and traditional knowledge emerged as an essential dimension of social prescribing, contributing to the reinforcement of self-esteem and identity among older adults.

Nurses mentioned various cultural and artistic activities, such as singing groups, sewing workshops, painting, gardening, cooking and crafts, which enable reconnection with meaningful experiences and collective memory. One participant said: ‘Activities linked to ancient times, such as singing, which are well received by some’ (Participant 10). Participant 11 added: ‘Older adults appreciate it when they are asked to teach traditional skills, such as sewing or cooking’. Participant 1 reinforced this perspective, emphasising that ‘Creative activities, such as painting or crafts, help them express what they feel and keep their minds active’. These testimonies illustrate how social prescribing can integrate identity and emotional dimensions, reinforcing a sense of belonging and cultural continuity. In this sense, social prescribing also acquires a dimension of restoring meaning and social recognition contributing to the appreciation of the role of older adults in the community.

### Contextual

The results point to structural barriers (especially transportation and limitations of time/organisational resources) and territorial facilitators (support from local authorities, senior universities and local associations) as determinants for the implementation and sustainability of social prescribing interventions.

#### Transportation barriers

The implementation of social prescribing interventions depends heavily on the organisational and community context. Among the limiting factors, transportation barriers were identified as the most common obstacle to adherence among older adults.

One participant stated: ‘Many older adults in rural areas find it difficult to travel to the city, which distances them from community initiatives’ (Participant 7). In the second group, Participant 15 reinforced this perception by stating that ‘transport difficulties prevent many from joining community initiatives’. This challenge is structural in nature, affecting mainly dispersed rural populations and compromising equity in access to community opportunities.

#### Available resources and time

The availability of resources and time within health units was identified as a key constraint on the implementation of social prescribing. In the FHUs, nurses reported organisational constraints that hinder the promotion of community initiatives. Participant 2 and Participant 14 both mentioned that consultations leave little time to promote such initiatives. Another participant offered a more critical perspective, stating that ‘sometimes, professionals themselves still place little value on this type of strategy’ (Participant 7). These accounts suggest that social prescribing remains undervalued among some professionals, highlighting the need for greater institutional recognition and organisational support for this practice to become established.

#### Support from local institutions

On the other hand, several community facilitators emerged, among which the support of local authorities, senior universities and local associations stands out. One of the participants mentioned ‘. . .initiatives by senior universities, promoted by city councils and even community gardens’ (Participant 13). Similarly, Participant 8 pointed out ‘. . .the support of local authorities and senior universities is essential to boost activities’. These community resources function as extensions of the healthcare system, strengthening the link between professional care and local resources and highlighting the potential of local partnerships for the sustainability of social prescribing interventions.

## Discussion

This study highlights the interdependence between therapeutic relationships, integrated care practices and contextual conditions, positioning social prescribing as a dynamic process within community-based care. These findings are consistent with the Fundamentals of Care Framework (Kitson et al., 2013; Feo and Kitson, 2016), which emphasises the interconnectedness of the therapeutic relationship; the integration of physical, emotional and social care; and the organisational context in which care is delivered. In line with previous research (Kitson et al., 2019), the findings suggest that fundamental care remains, in many contexts, insufficiently visible and integrated into routine practice. This study extends this perspective by demonstrating how social prescribing can strengthen and make visible fundamental care principles in PHC settings. From the nurses’ perspective, social prescribing emerges as a relational and integrated practice that depends on institutional and community contexts. The relational dimension is at the core of the process, sustaining motivation and adherence; the integration dimension expresses the articulation between physical, emotional and socio-cultural care; and the contextual dimension defines the conditions that enable or limit the practice. Together, these dimensions illustrate social prescribing as a dynamic and interdependent system of care within community settings.

These findings are also consistent with the literature, which highlights how the multifactorial needs of older adults are shaped by social determinants of health, including social isolation, living conditions and access to support networks (Al-Habbal et al., 2025; Sherman et al., 2024). However, they also point to ongoing challenges in translating these principles into routine practice, particularly in contexts where organisational constraints and limited resources restrict the implementation of integrated and person-centred approaches (Howarth et al., 2019; Khatri et al., 2023). Within this context, social prescribing emerges as an appropriate response to this complexity, as described in international approaches focused on the integration of care (Morse et al., 2022; Scarpetti et al., 2024). However, the present study extends this understanding by demonstrating how these processes are operationalised in everyday nursing practice. In particular, the findings show that nurses play a central role in translating the principles of social prescribing into concrete actions that promote engagement, autonomy and social participation among older adults. This is further supported by evidence indicating that active ageing is positively associated with well-being, perceived health and social participation and is influenced by factors such as health literacy and community involvement (Costa et al., 2023). In this sense, social prescribing, as operationalised by nurses in this study, emerges as a concrete mechanism for promoting this awareness by facilitating access to meaningful activities and encouraging the active involvement of older adults in the community.

### The relational dimension of social prescribing

The relational dimension identified in this study reinforces the central role of the nurse–older adult relationship as a foundation for effective social prescribing. Consistent with previous research, trust emerges as a key mechanism for fostering engagement and sustained participation in community-based activities (Husk et al., 2020; Younas et al., 2023). These findings align with evidence highlighting that nurse-led interventions in community settings rely heavily on trust-based relationships to support adherence and continuity of care (Thompson et al., 2026). This finding may be explained by the proximity and continuity of care inherent to nursing practice, which enable nurses to recognise emotional and social needs that often remain invisible within traditional biomedical models (Ribeiro et al., 2024).

However, the present study extends this understanding by illustrating how trust is not only a facilitator of engagement but also a critical response to the emotional and social vulnerabilities experienced by older adults. In particular, factors such as loneliness, grief and stress (Sadio et al., 2025; Sherman et al., 2024) reinforce the importance of the therapeutic relationship as an entry point for social prescribing interventions. In this context, trust becomes a key resource for mitigating psychosocial challenges and maintaining older adults’ connection with the healthcare system.

These findings further support the literature that identifies the relational dimension as a determinant of success in social prescribing, as engagement is strongly influenced by perceptions of support, empathy and respect (Carrera-Noguero et al., 2025; Costa et al., 2024). Therefore, the results of this study highlight the need to invest in professionals’ relational and communication skills, recognising them as core components of fundamental care (Feo and Kitson, 2016).

### Integrating physical, emotional and social care through social prescribing

The interventions identified in this study – namely group physical activities, health literacy initiatives and the promotion of local culture and traditions – illustrate an integrated approach to addressing the complex needs of older adults. This integration reflects the multifactorial nature of health, where physical, psychological and social well-being are closely interconnected (Frieden et al., 2023; Sadio et al., 2025). These findings are particularly relevant in the context of ageing, as loss of mobility and functionality makes older adults more dependent on interventions that promote autonomy and social participation (Ballmer and Gantschnig, 2024; Hafiz et al., 2024). In this sense, the combination of physical activity, health literacy and cultural practices represents a comprehensive response to these challenges.

Furthermore, the findings suggest that social prescribing extends beyond a purely biomedical approach, contributing to broader dimensions of well-being. Health literacy emerges as an important tool for empowerment and autonomy, enabling older adults to understand and manage their chronic conditions (Wilson et al., 2025). At the same time, engagement with cultural knowledge reinforces a sense of belonging and identity, aligning with evidence that points to the value of meaningful practices for healthy ageing and subjective well-being (Ballmer and Gantschnig, 2024; Mansell et al., 2024).

Importantly, these findings support existing evidence that social prescribing contributes to improved psychological well-being, social cohesion and community participation (Kurpas et al., 2023; Menhas et al., 2023; Morse et al., 2022). However, this study extends current knowledge by illustrating how these benefits are operationalised through nursing practice, highlighting the role of nurses in integrating multiple dimensions of care and supporting older adults as active agents within their communities.

### Contextual conditions shaping social prescribing in community nursing

The barriers identified in this study, including limited transportation, resource constraints and reduced consultation time, reflect constraints already described in the literature on the implementation of social prescribing in PHC (Simonelli et al., 2025; Spanos et al., 2025). These structural difficulties compromise equity in access, especially in rural areas, where isolation and limited availability of resources further increase the vulnerability of older adults (Al-Habbal et al., 2025). In line with previous research (Khatri et al., 2023), the findings highlight that continuity and coordination of care depend not only on organisational structures but also on the availability and articulation of local resources. When these conditions are fragile, the implementation of integrative practices such as social prescribing becomes more challenging.

However, the present study also highlights the importance of contextual facilitators, particularly local partnerships involving municipalities, senior universities and community organisations. These collaborations were identified as key elements supporting the sustainability of social prescribing initiatives. These results corroborate the conclusions of Fleming (2023) and Scarpetti et al. (2024), who argue that the sustainability of social prescribing depends on the creation of solid territorial networks and public policies that value intersectoral collaboration. Thus, the contextual dimension proves to be decisive not only in enabling interventions but also in ensuring their continuity and long-term impact. These findings suggest that, although social prescribing is widely promoted as an innovative and scalable approach, its implementation in practice remains uneven and highly dependent on local conditions, highlighting the need for structural and policy-level support (Morse et al., 2022; Scarpetti et al., 2024).

### Implications

This study reinforces the strategic role of nurses as mediators between the healthcare system and the community. Their proximity to patients and holistic view of the individual position them as key agents in the implementation of policies promoting active and healthy ageing (Roberti et al., 2025). However, the consolidation of social prescribing requires institutional support, formal recognition and dedicated time in clinical practice (Howarth et al., 2019). This need for institutional recognition and professional support is reinforced by evidence highlighting that nurses’ attitudes, competencies and workload conditions are critical for the successful implementation of community-based interventions, particularly within primary healthcare settings (Heumann et al., 2022; Miguéns et al., 2024; Sotelo-Daza et al., 2024).

The findings of this study also align with the growing body of literature advocating for the integration of community-based approaches into health systems as a means of addressing social determinants of health and promoting equity (Frieden et al., 2023; Morse et al., 2022). In particular, recent reviews on social prescribing in primary care emphasise the central role of nurses in bridging clinical care and community resources, facilitating access to non-medical interventions and supporting patient engagement (Husk et al., 2020; Morse et al., 2022). This perspective has been reinforced by international initiatives that demonstrate the benefits of social prescribing as a structuring practice in public health policies (Islam, 2025; Scarpetti et al., 2024).

In this context, recent analyses conducted in the study country and other European countries emphasise that, in the post-COVID-19 context, it is imperative to strengthen strategies for active and healthy ageing based on the community, the integration of care and intersectoral collaboration (Costa et al., 2021). The results of this study provide empirical support for these recommendations, showing that social prescribing, when integrated into PHC and supported by local partnerships, is a viable strategy for implementing active ageing policies at the territorial level.

From a policy perspective, it is essential to recognise social prescribing as a structural component of PHC, integrating it into national public health strategies. Such integration would enable a more effective response to the social determinants of health, contributing to the reduction of inequalities and the improvement in quality of life among ageing populations (Islam, 2025; Khatri et al., 2023).

Future research should examine the long-term outcomes of nurse-led social prescribing interventions and explore how contextual and organisational factors influence their implementation across different healthcare and cultural settings.

## Strengths and limitations

This study presents several strengths. It provides an in-depth qualitative exploration of nurses’ perspectives on social prescribing within PHC, an area that remains underexplored, particularly from a theoretically grounded perspective. The use of the Fundamentals of Care Framework offered a structured and comprehensive lens for analysing the relational, integrative and contextual dimensions of practice. Additionally, the use of focus groups enabled the collection of rich, experience-based data through interaction and reflection among participants.

However, some limitations should be considered. The study was conducted within a single regional health system, which may limit the transferability of the findings to other contexts. Furthermore, the use of focus groups may have influenced participants’ responses due to group dynamics or social desirability bias. Although data saturation was achieved, the relatively small sample size and the inclusion of only nurses may limit the breadth of perspectives, particularly those of other professionals and service users.

Future research should explore multiprofessional perspectives and include the views of older adults to better understand the impact of social prescribing interventions. Comparative studies across different geographical and organisational contexts would also contribute to a deeper understanding of how contextual factors influence the implementation and sustainability of social prescribing.

## Conclusion

This study shows that social prescribing, as perceived by PHC nurses, is a relational and integrated practice shaped by the territorial context. The findings demonstrate that the relational dimension, characterised by trust, motivation and continuity of care, is central to promoting older adults’ engagement and adherence to community-based interventions. At the same time, the integration of physical, emotional and socio-cultural care, implemented through interventions such as group physical activity, health literacy and engagement with local traditions, plays a key role in responding to the complex needs of this population.

The study also underscores the influence of contextual factors on the implementation and sustainability of social prescribing. Structural barriers, including transportation limitations, time constraints and limited organisational resources, may restrict access and hinder equity. Conversely, local partnerships with municipalities, senior universities and community organisations emerge as critical facilitators, supporting continuity of care and reinforcing the role of nurses as key mediators between health services and the community.

Overall, these findings contribute to a better understanding of how social prescribing is operationalised in everyday nursing practice, highlighting its potential to promote well-being, autonomy and social participation among older adults. To strengthen this approach, it is essential to invest in institutional support, professional training and the integration of social prescribing into public health strategies, ensuring more comprehensive and person-centred responses to the social determinants of health in ageing populations.

This study contributes novel evidence by demonstrating how the Fundamental Care Framework can be operationalised through nurse-led social prescribing practices in PHC, highlighting the relational and contextual dimensions that shape implementation in community settings.

**Key points for policy, practice and research**

- Social prescribing is a core community nursing practice, grounded in therapeutic relationships, integrated care and responsiveness to local contexts.
- The Fundamentals of Care Framework provides a robust lens for understanding how social prescribing addresses older adults’ physical, emotional and social needs in community settings.
- Trust, motivation and continuity of care are key mechanisms supporting older adults’ engagement in community-based activities, promoting autonomy and social participation.
- Organisational and contextual conditions strongly influence implementation, with barriers such as limited time and transportation constraints, and facilitators including partnerships with local authorities and community organisations.
- Health and social care policies should formally integrate social prescribing into primary healthcare, recognising and supporting the relational and contextual dimensions of community nursing practice.
- Future research should include the perspectives of older adults and multiprofessional teams and explore the long-term impact of social prescribing across different contexts.
- This study provides original qualitative evidence on how nurses operationalise social prescribing within the relational, integrative and contextual dimensions of the Fundamental Care Framework.

## Supplemental Material

sj-docx-1-jrn-10.1177_17449871261458631 – Supplemental material for Social prescribing interventions in response to older adults’ needs in community settings – nurses’ perspectives: a qualitative study Supplemental material, sj-docx-1-jrn-10.1177_17449871261458631 for Social prescribing interventions in response to older adults’ needs in community settings – nurses’ perspectives: a qualitative study by Rute Sadio, Adriana Henriques, Paulo Nogueira and Andreia Costa in Journal of Research in Nursing
