# Supplementary material for: Social prescribing interventions in response to older adults’ needs in community settings – nurses’ perspectives: a qualitative study
Source: J Res Nurs. 2026 Aug 5:17449871261458631. Online ahead of print. doi: 10.1177/17449871261458631 (PMC13442910; doi:10.1177/17449871261458631)
Supplement: Supplementary file 1 [file sj-docx-1-jrn-10.1177_17449871261458631.docx]

**COREQ Checklist – Consolidated Criteria for Reporting Qualitative Research**

Social prescribing interventions in response to older adults’ needs in community settings: nurses’ perspectives

**Domain 1. Research team and reflexivity**

1. Interviewer/facilitator

Location: Methods – Data collection

Details: Focus groups were moderated by the principal investigator.

2. Credentials

Location: Methods – Study design and theoretical framework

Details: Principal investigator was a nurse and doctoral student.

3. Occupation

Location: Methods – Study design and theoretical framework

Details: Primary health care nurse.

4. Gender

Location: Methods – Study design and theoretical framework

Details: Female researcher.

5. Experience and training

Location: Methods – Study design and theoretical framework

Details: The principal investigator had previous experience in primary health care and qualitative research and received supervision from senior researchers.

6. Relationship established

Location: Methods – Data collection

Details: The researcher acknowledged a prior professional relationship with participants as colleagues in primary health care.

7. Participant knowledge of the interviewer

Location: Methods – Data collection

Details: Participants were informed about the study objectives and the researcher’s role before participation.

8. Interviewer characteristics

Location: Methods – Rigor, reflexivity and ethical considerations

Details: Reflexivity was addressed through reflective records, allowing identification and mitigation of potential biases related to the researcher’s professional experience.

**Domain 2. Study design**

9. Methodological orientation and theory

Location: Methods – Study design and theoretical framework

Details: Qualitative descriptive approach informed by a social constructionist perspective.

10. Sampling

Location: Methods – Participants

Details: Intentional (purposive) sampling.

11. Method of approach

Location: Methods – Participants

Details: Participants were recruited through institutional contacts within the health units and invited by email.

12. Sample size

Location: Methods – Participants

Details: Eighteen primary health care nurses participated in two focus groups.

13. Non-participation

Location: Methods – Participants

Details: Eighteen nurses were invited and all agreed to participate; there were no refusals or withdrawals.

14. Setting of data collection

Location: Methods – Data collection

Details: One focus group was conducted face-to-face at a primary health care facility and the second online in a private setting.

15. Presence of non-participants

Location: Methods – Data collection

Details: Only the researcher and participants were present during the focus groups.

16. Description of sample

Location: Methods – Participants; Table 1

Details: Participants’ gender, nursing specialty, years of professional experience and workplace were described.

17. Interview guide

Location: Methods – Data collection; Table 2

Details: A semi-structured interview guide based on the literature was developed, pre-tested with two PHC nurses and minor adjustments were made.

18. Repeat interviews

Location: Methods – Data collection

Details: No repeat interviews were conducted.

19. Audio/visual recording

Location: Methods – Data collection

Details: Focus groups were audio-recorded and transcribed verbatim.

20. Field notes

Location: Methods – Data collection

Details: Field notes documenting relevant non-verbal elements and researcher observations were prepared after each focus group.

21. Duration

Location: Methods – Data collection

Details: Each focus group lasted approximately 90 minutes.

22. Data saturation

Location: Methods – Data analysis

Details: Data saturation was achieved after the second focus group, with no new relevant information emerging.

23. Transcripts returned

Location: Methods – Data collection

Details: Transcripts were returned to participants (member checking), who confirmed their accuracy.

**Domain 3. Analysis and findings**

24. Number of data coders

Location: Methods – Data analysis

Details: Initial coding was performed by the principal investigator and subsequently reviewed and validated by the co-authors.

25. Description of the coding tree

Location: Results; Table 3

Details: Themes, dimensions and subthemes were organised according to the Fundamentals of Care Framework.

26. Derivation of themes

Location: Methods – Data analysis

Details: Themes were developed using a deductive framework-informed analysis while allowing inductive identification of emerging themes.

27. Software

Location: Methods – Data analysis

Details: MAXQDA software was used.

28. Participant checking

Location: Methods – Data collection

Details: Participants confirmed the accuracy of the transcripts through member checking.

29. Quotations presented

Location: Results

Details: Verbatim quotations are presented with participant identifiers to support each theme.

30. Data and findings consistent

Location: Results and Discussion

Details: Findings are supported by quotations and interpreted consistently within the Fundamentals of Care Framework.

31. Clarity of major themes

Location: Results

Details: Major themes are clearly presented according to the Relational, Integration and Contextual dimensions.

32. Clarity of minor themes

Location: Results

Details: Subthemes are explicitly described and supported by participant quotations.
